# Supplementary material for: StructuralDPPIV: a novel deep learning model based on atom structure for predicting dipeptidyl peptidase-IV inhibitory peptides
Source: Bioinformatics. 2024 Feb 1;40(2):btae057. doi: 10.1093/bioinformatics/btae057 (PMC10904144; doi:10.1093/bioinformatics/btae057)
Supplement: btae057_Supplementary_Data [file btae057_supplementary_data.docx]

# Supplementary Materials

| Modal fusion method  Batch size or filter numbers | ⨁* | Concat and ⨁ |
| --- | --- | --- |
| BS = 16, FN = 10 | 0.9098 | 0.9139 |
| BS = 32, FN = 10 | 0.9135 | 0.9101 |
| BS = 64, FN = 10* | 0.9098 | 0.9060 |
| BS = 128, FN = 10 | 0.9135 | 0.9098 |
| BS = 32, FN = 90 | 0.9098 | 0.9060 |

**Supplementary Table 1**. Average Model performance (accuracy) with hyperparameters changes, Where BS means batch size, FN means filter number in the TextCNN module, ⨁ means element-wise multiplication on vector. Hyperparameters with ***** is the configuration used when StructuralDPPIV Manuscript was originally submitted.

| Fold Number | Accuracy on Test Set |
| --- | --- |
| 1 | 0.8692 |
| 2 | 0.9065 |
| 3 | 0.8692 |
| 4 | 0.8868 |
| 5 | 0.8962 |
| 6 | 0.9528 |
| 7 | 0.8679 |
| 8 | 0.8679 |
| 9 | 0.9151 |
| 10 | 0.9151 |
| Average of StructuralDPPIV | 0.8947 |
| Average of StackDPPIV | 0.8890 |

**Supplementary Table 2**. We divided the original training dataset into 10 non-overlapping parts. In each fold, we trained the model on nine parts and used the remaining part as a validation set. After training, we selected the model checkpoint that performed best on the validation set as the performance record of this fold. The model's hyperparameters were kept consistent with the previous version. The same ablation experiment was previously conducted in StackDPPIV (Phasit, et al., 2022). This work reported an average accuracy of 0.8890 across ten folds.


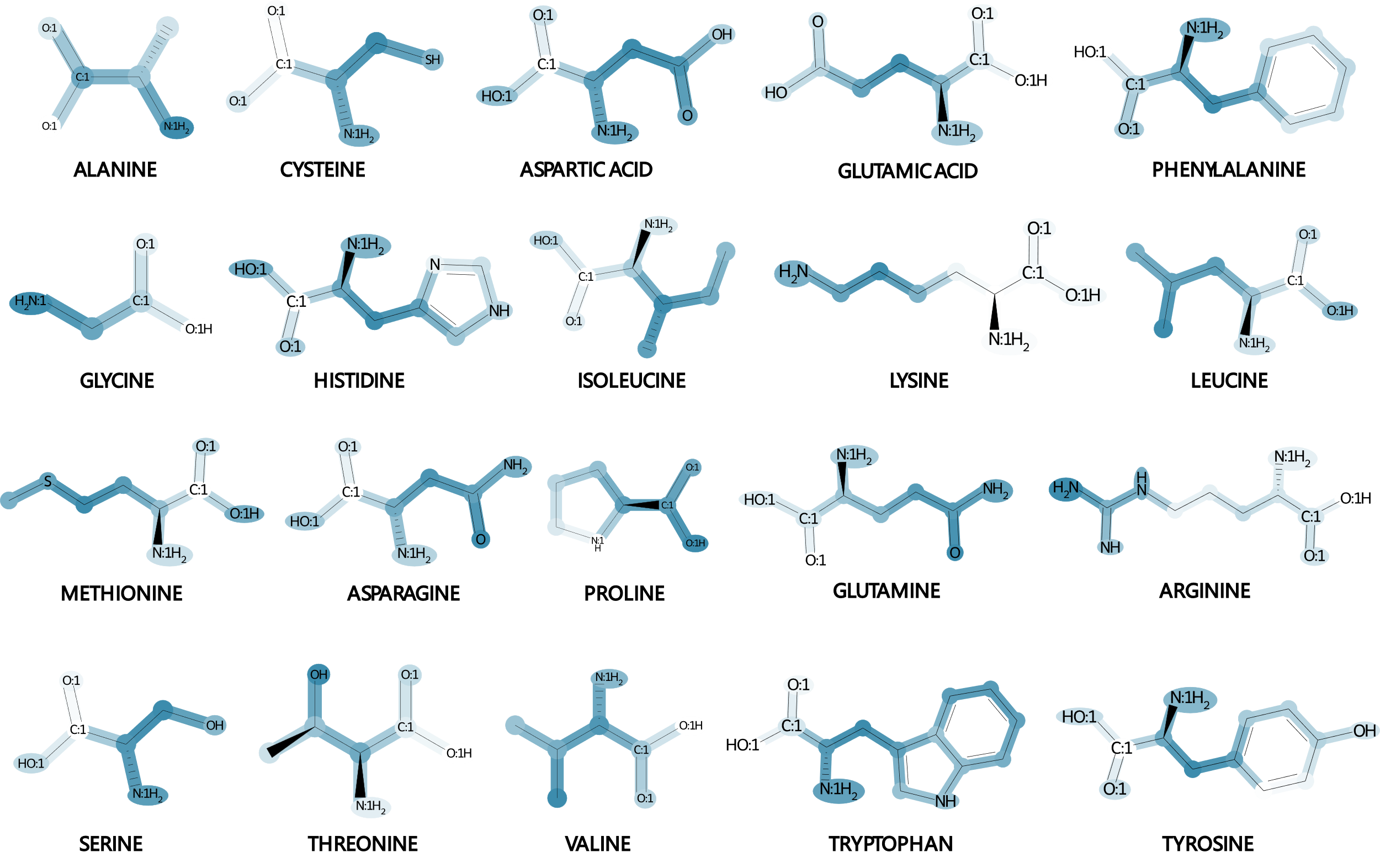


**Supplementary Figure 1.** Cam analysis plots of 20 kinds of amino acids.
